# Asian elephants (*Elephas maximus*) demonstrate inhibitory control in a detour task

**DOI:** 10.1371/journal.pone.0356549

**Published:** 2026-09-09

**Authors:** Sydney F. Hope, Sarah L. Jacobson, Sangpa Dittakul, Gillian Schmitt, Mananya Pla-ard, Marnoch Yindee, Taweepoke Angkawanish, Joshua M. Plotnik

**Affiliations:** 1 Department of Psychology, Hunter College, City University of New York, New York, New York, United States of America; 2 Psychology Ph.D. Program, CUNY Graduate Center, City University of New York, New York, New York, United States of America; 3 Department of Psychology, Saint Xavier University, Chicago, Illinois, United States of America; 4 Golden Triangle Asian Elephant Foundation, Chiang Saen, Chiang Rai, Thailand; 5 Akkhraratchakumari Veterinary College, Walailak University, Nakhon Si Thammarat, Thailand; 6 National Elephant Institute, Forest Industry Organization, Lampang, Thailand; University of Mississippi School of Pharmacy, UNITED STATES OF AMERICA

## Abstract

Inhibitory control—or the suppression of an impulsive, or prepotent, response in favor of a less obvious, but more effective behavior—is an important aspect of executive function, decision-making, and cognitive flexibility. Although elephants have large brains and behave flexibly, little is known about their capacity for inhibitory control, which limits our understanding of its evolution across species. We tested 16 (8M, 8F, 4–60 yrs) Asian elephants (*Elephas maximus*) at the National Elephant Institute in Thailand using a classic detour paradigm. Elephants first learned to reach their trunk around an opaque box to obtain food from a single opening. We then presented them with a transparent box with front-facing holes, where subjects needed to continue to reach around the box while inhibiting the impulse to reach straight toward the visible food and front-facing odor cues. We found that elephants performed similarly when detouring around both opaque and transparent boxes. This may indicate a strong ability to inhibit the impulse to reach straight forward, but may also indicate that the transparent box did not produce cues strong enough to elicit a prepotent response. In addition, we conducted trials in which we changed the box orientation so that elephants needed to inhibit using a previously learned response (i.e., a direction in which they had previously detoured) in favor of detouring in the newly changed direction. Here, we found evidence for a strong prepotent response: elephants’ latency to detour around the box significantly increased when the box orientation changed. However, detour performance quickly improved across trials in which the orientation was constant, indicating effective inhibition of the previously learned response. Interestingly, performance in these trials worsened with age, revealing potential evidence for cognitive decline. This study provides some of the first evidence for inhibitory control in this species, and furthers our understanding of convergent cognitive evolution across distantly related taxa.

## Introduction

Inhibitory control—or the ability to suppress a dominant, but counterproductive, response in favor of an appropriate behavior—is a core executive function that is important for self-control, decision-making, and cognitive flexibility [1]. In humans, greater inhibitory control is related to a greater propensity to cooperate [2], higher proficiency in mathematics [3], and better reading comprehension [4]. In non-human animals, interspecies differences in inhibitory control have been linked to social complexity [5], absolute brain volume [6, but see 7, which found that corvids had similar inhibitory control to great apes], and dietary complexity [6]. Furthermore, variation within a species correlates with predictors of fitness. For example, male song sparrows with greater inhibitory control also have a larger song repertoire size—a trait preferred by females [8]—and female Australian magpies with greater inhibitory control (along with other aspects of cognition) have greater reproductive success [9].

One classic method to test inhibitory control is to use the detour paradigm [reviewed in 10], where an individual must detour around a transparent barrier to obtain a reward. In many studies [e.g., 6,11,12], subjects are first trained to detour around an opaque barrier before being presented with a similar, but transparent, barrier. In this way, any reduction in performance when interacting with the transparent barrier suggests a ‘knowing/acting mismatch’—where the individual knows the correct detour strategy, but acts incorrectly due to the impulse to reach straight toward the visible goal [1,10]. However, after multiple trials using this method, subjects may perform well not because they are inhibiting an impulse, but instead because they have overlearned the strategy. Overcoming this habitual motor response also requires inhibitory control [1]. To investigate inhibitory control performance in relation to an overlearned motor response, other studies have varied the direction in which the animal must detour around the transparent barrier [e.g., 13,14]. In this way, the animal must continue to inhibit the impulse to reach toward the visible reward, while also inhibiting any overlearned motor responses and flexibly shifting their behavior to adapt to the changing directions.

The detour paradigm has been tested across at least 96 species [10] and has been shown to be a useful tool for studying the evolution of inhibitory control, despite recent studies that have questioned its validity. For example, performance in detour tasks may also be related to previous experience with transparent barriers [15], behavioral traits such as persistence [15], personality traits such as proactivity, boldness, and exploration [16–18], factors related to motivation such as body condition [19], and other cognitive processes such as prior learning of a fixed motor response [20]. In addition, some studies find that performance in detour tasks is not related to performance in other assays classically used to measure inhibitory control (e.g., the A-not-B task) [21,22]. Together, these findings may suggest that detour tasks measure non-target cognitive (e.g., learning) or non-cognitive (e.g., motivation, personality) traits, instead of inhibitory control [for reviews see 23,24]. However, other studies do find that performance in detour tasks is repeatable across different assays—within [12] and across species [6]—and is unrelated to other potentially confounding traits [12,25–27], suggesting that the detour task may indeed be an appropriate measure of inhibitory control. Importantly, considerations made during experimental design and analysis can improve the validity of detour tasks [23]. For example, one important aspect of an inhibitory control task is that it elicits a ‘characteristic error pattern’, where animals first exhibit an impulsive response before improving [23]. In addition, sources of variation—such as motivation and previous experience with transparent surfaces—should be controlled for when possible [23]. Control variables—such as age—should also be included in analyses [23]. Finally, repeatability of individual performance should be considered to ensure that the task reliably measures the same behavioral endpoint over time [23]. Therefore, by carefully considering these factors during experimental design and analyses, the detour task can remain an appropriate measure of inhibitory control and a valuable task to investigate convergent cognitive evolution due to its simplicity and widespread usage across species.

One taxon in which the detour task has never been tested is the elephant. Asian elephants (*Elephas maximus*) in particular are large-brained mammals living in complex, fission-fusion social groups [28], that make flexible decisions based on changing environmental pressures [29,30], and can solve problems both innovatively [31–33] and cooperatively [34,35]. All of these traits are related to a strong capacity for inhibitory control in other species [2,5,6,36–38], suggesting that elephants should also exhibit an ability to inhibit their behavior. However, the single study to-date that has investigated inhibitory control in Asian elephants found that they performed poorly on the A-not-B task—a task where animals must inhibit choosing a previously rewarded location [6]. Therefore, based on current knowledge, elephants stand out as having particularly poor inhibitory control in relation to their brain size [6,23]. However, to succeed in the A-not-B task, the animal must watch an experimenter as they move the reward from the previously rewarded location (i.e., A) to the new location (i.e., B). Because Asian elephants rely more on their senses of smell, hearing, and touch compared to vision [39], and have already been shown not to follow human social cues such as pointing [40,41], a task that required subjects to inhibit action in response to watching human behavior may not have been appropriate for elephants.

The elephant taxon may in fact be a good model to investigate differences in inhibitory control between sexes and across ages, if a task with appropriate ecological validity can be implemented. Females live in fission-fusion societies, and cooperate to care for offspring, find resources, and defend the herd from predators [42–44]. In contrast, males leave family groups when they reach sexual maturity, to live solitarily or among other males [45,46]. Because living socially—specifically, both acting cooperatively [2,37] and living in fission-fusion social groups that are fluid in size and change based on resource abundance or other ecological factors [5]—has been linked to greater inhibitory control, we may expect female elephants to have higher inhibitory control compared to males. Furthermore, elephants are long-lived, with lifespans of >60 years [47]. Although, across taxa, inhibitory control generally follows an inverted U-shaped curve where performance increases during early development and decreases in old age [21,48–50], elephants may be different. For example, despite their longevity, cognition does not appear to decline in older elephants [47]. In fact, older African savannah elephant (*Loxodonta africana*) matriarchs appear to have the most social and ecological knowledge within a herd [42,51]. Therefore, we may not expect a cognitive decline in old elephants.

Here, we aimed to describe inhibitory control in Asian elephants using the classic detour paradigm, with methodological adaptations for elephants. In our study, elephants first learned to reach their trunk around an opaque box to obtain food from a single opening. Then, they were confronted with a transparent box with holes in the front (to release odor cues), where they needed to inhibit the impulse to reach straight toward the food reward that they could see and smell from the front [27], and instead detour around the box as they had learned with the opaque box. Then, to determine whether elephants could inhibit a previously learned motor response, we changed the orientation of the box so that the opening was facing upward, downward, left, or right, and elephants needed to flexibly change their behavior to find the opening. In both variations, we measured task performance as the latency for elephants to enter their trunk into the opening, starting from the first time they touched the box. In addition, we measured the amount of time elephants’ trunks spent inside the box before they obtained the food, as a measure of motor control or, potentially, exploratory behavior. Similar to other species [e.g., 11,52–54], we predicted that, when elephants were first presented with the transparent box, they would be slower to detour compared to when interacting with the opaque box. We also expected that, when the orientation of the box was changed, elephants would be slower to detour compared to previous trials in which they were presented with an orientation they had previously learned. We also expected that inhibitory control would vary by sex and across ages. Specifically, we predicted that females would have more inhibitory control—or would exhibit shorter latencies to enter the box and obtain the food—than males due to their social structure [46] and because females have greater inhibitory control in other species [55–60]. Our investigation of the relationship between inhibitory control and age was exploratory because there were two plausible but opposing predictions. First, inhibitory control may decline with age, as in other species [21,48–50]. Alternatively, unlike other species, elephants might not succumb to cognitive decline [47], and inhibitory control may persist across age groups. Finally, regardless of sex or age, we predicted that all individuals would show evidence of learning, where inhibitory control performance would improve across successive trials where the box was consistent in opacity and orientation [10].

## Materials and methods

### Subjects

For the current study, we worked with 16 Asian elephants (8 males and 8 females; ages 4–60 years) at the National Elephant Institute (NEI) in Lampang, Thailand from August 2023 – January 2024. The NEI is an elephant health and conservation facility run by the Thai government. It has the largest elephant hospital in Thailand, with a team of more than ten full-time veterinarians that provides free veterinary care to any elephant in the country. In addition, each elephant living at the NEI has a mahout, or caretaker, who is responsible for daily care and training. Elephants in this study followed their normal feeding schedule. Although it is hypothesized that inhibitory control may be hindered due to hunger [19,23], we think this is unlikely to be a factor in this study because feeding habits remained unchanged. None of the elephants that participated in this study had previously been exposed to similar cognitive tests involving reaching around a transparent barrier to gain access to food, and likely none had experience with transparent materials. Therefore, we did not expect that experience with transparent materials would affect the results of this experiment [e.g., 15]. Some of the elephants also participated in an experiment investigating future planning and collective intelligence shortly before or concurrently with this study [61], and some had participated in an experiment investigating cooperation more than 15 years prior [35]. However, these experiments used completely different apparatuses and tested different aspects of cognition, and thus should not have affected the results of the current experiment. Demographic information can be found in Table A in S1 Appendix. We attempted to work with one additional female (64 yrs) but stopped working with her due to her inability to complete the task and her disinterest in participating; she did not complete any trials.

### Ethics statement

The research protocol was reviewed and approved by Hunter College (JP_ProblemSolvingElephants 6/26) and Walailak University (WU-ACUC-66042) Institutional Animal Care and Use Committees. This research was also approved by the National Research Council of Thailand and the National Elephant Institute’s administrative and veterinary teams.

### Experimental apparatuses and setup

Elephants detoured around two different barriers—a familiarization box and a test box—which were adapted from similar detour task experiments [e.g., 11,14]. Both boxes (40 cm x 40 cm x 40 cm) were made from five 5-mm thick acrylic panels, reinforced with metal edges, and had one open side. The familiarization box was spray-painted black on the outside so that it was opaque. The test box differed from the familiarization box in two ways: it was transparent and it had five holes (2 cm diameter) in the front side. This allowed elephants to both see the food and smell it from the front. Two blunt metal dowels were secured in the inside of each box, in the center of the back panel, so that the food reward could be attached to the inside of the box. We mounted the boxes on a tree at each elephants’ eye level (i.e., box height differed based on elephant height) and manipulated them as needed so that the orientation of the open side could either be on the top, bottom, left, or right. In each of these orientations, the holes of the test box were always in the front panel. We baited the boxes with a high-value food reward—either a slice of pineapple, half of an apple, or one small banana; the food type was chosen per elephant based on consultation with the mahouts and veterinarians. We recorded all trials with at least two video cameras (Sony models FDR-AX100, HDR-CX405 and/or GoPro model HERO8 Black) set up on tripods, to record left and right angles of the box. We tested elephants individually and in an area that was away from other elephants, although occasionally another elephant who was being tested on the same day waited with their mahout in the vicinity. In these cases, we asked the mahout to have the elephant turn their back to the box and to ensure that they did not interact with the box or the elephant who was being tested.

Each elephant participated in four sessions of testing. The first two sessions tested whether elephants could inhibit the impulse to reach straight toward the front-facing visual/olfactory stimuli and instead continue to perform the detour that they had learned (i.e., overcome the knowing/acting mismatch). The last two sessions tested whether elephants could flexibly change the direction by which they detour, instead of continuing to use a learned strategy after it was no longer effective (i.e., inhibit a previously learned response). The minimum time between different sessions was 3 hours (i.e., one session in the morning and the next in the afternoon of the same day) and the maximum time was 3 days; these differences were due to variation in the mahouts’ and elephants’ daily schedules that were beyond the researchers’ control. After an elephant completed a session, we cleaned all boxes with isopropyl alcohol—to remove any olfactory cues—before using them with any other elephants.

### Overcoming the knowing/acting mismatch – sessions 1 and 2

**Fig 1:**
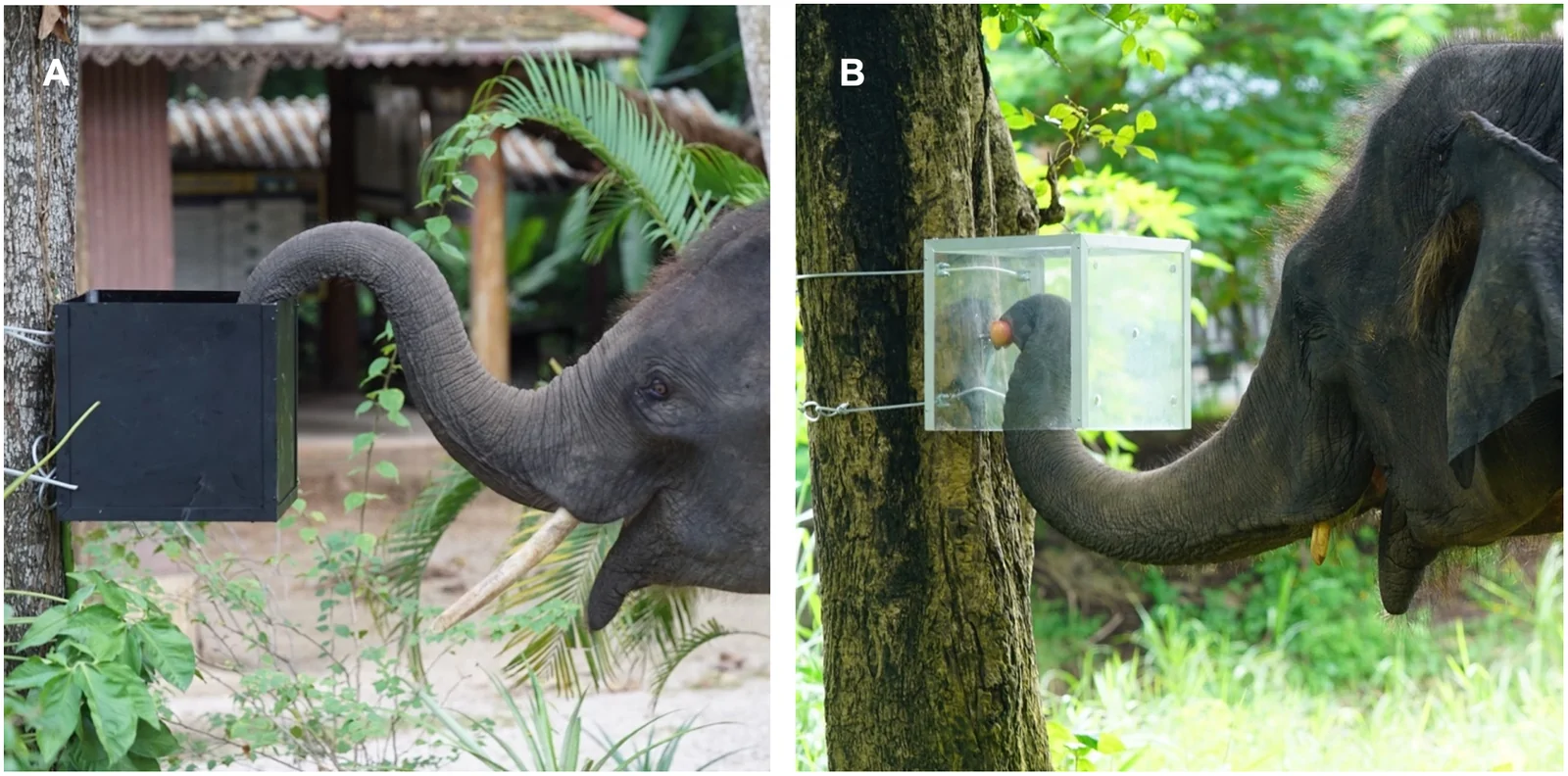
Experiment Setup. (A) An elephant enters the “over” opening to gain access to food in a familiarization trial. (B) An elephant enters the “under” opening to gain access to food (apple) in a test trial. Photos were cropped to remove extraneous background material; the left image was mirrored. Photos by Sangpa Dittakul.

We tested inhibitory control in the context of the knowing/acting mismatch twice over two sessions [62]. Each session consisted of four familiarization trials [6,15] followed by four test trials. During the familiarization trials, elephants learned how to detour their trunk around the familiarization (i.e., opaque) box and into the opening to reach the food (Fig 1A; S1 Video). In the test trials, elephants were then confronted with the test box (i.e., transparent with holes; Fig 1B; S2 Video). The test trials should have been more difficult because, to reach the food reward, elephants needed to inhibit the impulse to reach straight toward the food that they could see and smell in front of them and, instead, continue to detour around the box in the way that they had learned during the familiarization trials. We oriented the boxes either in the “over” orientation (opening at the top) or the “under” orientation (opening at the bottom). Within the same session, the familiarization box and test box were always in the same orientation, and the orientation was the opposite in the following session. The orientation that an elephant received first was counterbalanced across sexes and ages.

Within a session, test trials began immediately after elephants completed the four familiarization trials. However, if the elephant did not successfully obtain the food in under three minutes in at least three familiarization trials, they then repeated four additional familiarization trials before moving to test trials. This was necessary for two elephants; to meet these criteria, one female (58 yrs) completed two sets of familiarization trials, and one male (60 yrs) completed three sets. In these cases, only the final set of familiarization trials (i.e., in which the elephant met criteria) was included in analyses. The familiarization trials may have also helped to habituate any potentially shy or neophobic elephants to the novelty of the apparatus, food, or location. This should have reduced any potential relationship between elephant personality and inhibitory control performance [16–18].

### Inhibition of a previously learned response – sessions 3 and 4

We tested elephants’ abilities to inhibit a previously learned response over two sessions. We used the test box for all trials. The first session (session 3) consisted of 16 trials. First, we oriented the test box in either the left or right orientation for four consecutive trials; the orientation with which elephants began was counterbalanced across sexes and ages. We then oriented the box to the other side (i.e., either left or right) for four trials, followed by four trials of either the over or under orientation (counterbalanced across sexes and ages), and four trials in the final orientation (i.e., either over or under). This tested the elephants’ abilities to learn how to obtain the food when faced with a novel box orientation across four trials, and subsequently the speed at which they could inhibit a previous response and learn a new response when the orientation changed (S3 Video). The next session (session 4) consisted of eight trials, where the orientation order was pseudorandomized so that the same orientation was never used consecutively, and each orientation (left, right, over, under) was used twice. This tested the elephants’ flexibility in responses when conditions were constantly changing.

Furthermore, the first trial of the third session also served as a control to determine whether elephants may have used odor cues to solve the task. It is possible that, instead of learning how to detour around the box in sessions 1 and 2, elephants may have simply used the direction from which the strongest odor cues were emitted to find the opening in each trial. However, in the first trial of session 3, we presented elephants with the test box oriented in a way in which they had never experienced (i.e., left or right). Therefore, if elephants used solely odor cues to find the opening of the box, we would expect all elephants to go straight to the opening in this trial. However, if they did not go straight to the opening, it would suggest that they did not detour based solely on odor.

### Experimental procedure

We used the same procedure for all trials of all sessions. First, elephants stood with their mahout at ~10 m from the box. Then, one experimenter baited the box while another blocked the elephants’ view of the box by holding up an opaque sheet. When ready, the experimenters informed the mahout, who then told the elephant to walk toward the box (*bpai*— “go” in the Thai language). When any part of the elephant’s body passed a spot that was marked 7 m from the box, the experimenter lowered the opaque sheet and walked away, and the trial began. Elephants started at a distance, allowing them time to approach the box and potentially detect visual or olfactory cues from afar. This also allowed them the opportunity to localize the opening without tactile cues, if possible, when box orientations were switched in sessions 3 and 4. The trial ended when the elephant ate the food, and the mahout called the elephant back. Trials followed immediately one after another until the session was complete. Mahouts fed elephants sunflower seeds between trials as necessary to keep them motivated to stay in the general area of testing. Five of the 16 elephants were tethered during testing (a common husbandry practice at NEI for all of the elephants), but the length of the tether did not impact the elephants’ ability to interact with the box in any way.

Mahout involvement depended on the trial. In the first familiarization trial of session 1, mahouts could give the verbal commands of *bpai* (go forward), *sok* (walk backward), *maa* (come), and *bon* (put trunk up) to guide them to the opening, as well as *koi koi* (be gentle) if the elephant attempted to break the box. Because, in this trial, elephants first needed to learn that the box contained food, mahouts and experimenters were also allowed to approach the box and gesture toward it to encourage the elephant to interact with the box, if needed. If the elephant had not acquired the food after 10 min, the mahouts or experimenters then used a piece of food to guide the elephant’s trunk into the box. This extra step was necessary for three elephants (one male, 60 yrs; two females, 41 and 58 yrs). Because this single trial differed from all others, we excluded it from all analyses for all elephants. In all other familiarization trials, mahouts and experimenters stood away from the box and did not make any gestures; however, mahouts were allowed to say *bpai* (go forward), *sok* (walk backward), and *maa* (come) to tell the elephant to move toward the box, *koi koi* (be gentle) if the elephant attempted to break the box, and *bon* (put trunk up) if the elephant was standing near the box but not interacting with it. In all trials in which the test box was used, mahouts could only say *bpai* (go forward), *sok* (walk backward), or *maa* (come) to tell the elephant to go toward the box. Once the elephant began interacting with the box, they were asked to refrain from all verbal commands, except *koi koi* (be gentle) if the elephant attempted to break the box.

### Behavioral coding

We coded behavior from videos using BORIS [63]. For each trial, we recorded each time that the elephant touched the box, when their trunk entered the box, and when their trunk exited the box. We defined a ‘touch’ as when any part of the trunk contacted the outside of the box. A touch ended either when the trunk was no longer in contact with the box or if the trunk slid to a different side of the box; in the latter case, we coded this as a separate ‘touch’ behavior. We defined ‘entering the box’ as when the tip of the trunk entered the open side of the box. We defined ‘exiting the box’ as when the tip of the trunk exited the box and remained outside the box for more than two seconds. It was possible for an elephant to enter and exit the box multiple times before the end of the trial (i.e., before they ate the food). For each trial, we then calculated the number of times that the elephant touched the box before they entered the open side of the box for the first time (hereafter, number of touches), the total amount of time (sec) that the elephants’ trunk was inside of the box before the trial ended (hereafter, time spent inside the box), and the latency to enter the box (calculated from the time that the elephant first touched the box). If the elephant reached directly into the open side without touching the box, the latency to enter was zero. Latency to enter and number of touches are classic measures of success for inhibitory control tasks [e.g., 11,14] and indicate the time and energy it takes for the individual to successfully detour around the barrier. In addition, the time spent inside the box indicates the time individuals needed to succeed at the task (i.e., obtain the food) once they had already successfully detoured, which may be related to motor control (i.e., pinpointing and grasping the food once their trunk is inside the box) or exploratory behavior (e.g., searching inside the box for more food).

All behaviors were coded by co-author G.S. To assess inter-rater reliability, an independent observer who had no prior experience with the experiment coded 25% of the trials (*N* = 160 trials). Videos were chosen pseudo-randomly, to ensure an even distribution of subjects, sexes, ages, and session types. Both coders were blind to the age and sex (if not evident from physical features) of the elephants.

### Statistical analyses

All statistical analyses were conducted using R statistical software version 4.5.2 [64] using the following packages: *lme4* [65], *car* [66], *dplyr* [67], *plyr* [68], and we created figures using *ggplot2* [69] and *ggthemes* [70]. Number of touches was highly correlated with latency to enter the box (*r* = 0.79, *p* < 0.001) and, thus, we excluded number of touches from our analyses to reduce redundancy. We used simple linear or linear mixed effect models, and we investigated histograms of residuals, normal quantile plots, and fixed vs. residual plots to ensure that assumptions of each model were met. For each model described below, we ran one version with ‘latency to enter’ and another with ‘time spent inside the box’ as the dependent variable. Because all models were conducted twice (i.e., with two dependent variables), we used a Bonferroni correction and set significance at α = (0.05/2) = 0.025. Dependent variables were always log-transformed to meet model assumptions. The factors and data included in all models, along with model results, are reported in Table B in S1 Appendix. Because length of time between sessions ranged from 3 hours to 3 days (*see Experimental apparatuses and setup*) we originally included ‘time delay since last session’ (0–3 days, with dummy-coding of 4 for session 1) as a random effect in all models. However, this did not qualitatively change the significance of any of our main independent variables of interest, and resulted in multiple ‘singular’ errors—suggesting that little to no variation was explained by the random effect—so we decided to exclude it from all models.

#### Prepotent responses.

One important aspect of inhibitory control tasks is that they elicit a prepotent response—or impulse—that is subsequently inhibited based on an individual’s inhibitory control [23]. To determine whether our tasks elicited a prepotent response (Table 1), we first investigated whether behaviors differed between the last familiarization trial (i.e., after they had learned the detour strategy during four trials) and the first test trial (i.e., when they were first confronted with the transparent surface) in sessions 1 and 2 (Table B in S1 Appendix, Question 1). We also investigated whether changing the box orientation elicited a prepotent response (Table B in S1 Appendix, Question 2) by determining whether behaviors differed between trials in which the orientation was initially changed in session 3 (i.e., trials 1, 5, 9, and 13) and the trial immediately before the orientation change (i.e., after they had learned the previous orientation). We used linear mixed effects models with trial type (i.e., familiarization vs. test or before vs. after box orientation change) as the predictor and elephant ID as a random effect because trials were repeated on the same individuals. Models investigating data from sessions 1 and 2 also included session number as a covariate, and models investigating data from session 3 included ‘orientation change number’—or the number of times the box orientation changed (1–4)—as a covariate.

**Table 1:** Testing the Prepotent Response.

Session	Trials	Box used	Box orientation	Main trial numbers to test for prepotent response	Predicted error pattern	Behavior if prepotent response is inhibited
1 and 2	Familiarization 1–4	Familiarization (opaque)	Over or Under (counterbalanced between session 1 and 2 across elephants)		–	–
1 and 2	Test 1–4	Test (transparent)	Over or Under (same orientation as 4 familiarization trials)	Familiarization 4 vs. Test 1	Slower latency to enter the opening in test compared to familiarization; does *not* continue to perform behavior learned during familiarization (i.e., directly reaching over or under)	Directly or quickly enters opening (i.e., performs behavior learned during familiarization)
3	1-4	Test (transparent)	Left or Right (counterbalanced)	Session 2 Test 4 vs. Session 3 Trial 1	Slower latency to enter the opening when box changes to a new orientation—one that is different from that which was learned previously	Directly or quickly enters opening in novel orientation
3	5-8	Test (transparent)	Left or Right (opposite of previous 4 trials)	Trial 4 vs. Trial 5		
3	9-12	Test (transparent)	Over or Under (counterbalanced)	Trial 8 vs. Trial 9		
3	13-16	Test (transparent)	Over or Under (opposite of previous 4 trials)	Trial 12 vs. Trial 13		

Sessions 1, 2, and 3 were used to investigate whether elephants exhibit and/or can inhibit a prepotent response. Session numbers, trial numbers, and boxes used (transparency and orientation) are listed. For each session, we also specify the trials that were compared when testing for a prepotent response, along with the predicted behavioral response if elephants exhibited (i.e., predicted error pattern) and/or inhibited the prepotent response.

#### Repeatability.

Another important aspect of inhibitory control tasks is that responses are repeatable within individuals [23]. To measure repeatability within individuals, we used the *rpt* function from the *rptR* package [71] with trial number and session (when appropriate) as fixed factors and elephant ID as the random effect, and we log-transformed dependent variables. We only included trials in which the box was transparent, and calculated repeatability of data from sessions 1 and 2 separately from session 3, and session 4. We calculated *p*-values using permutation tests (1000 permutations).

#### Age and sex differences.

Next, we investigated whether inhibitory control differed between sexes or across ages. To examine prepotent responses without any effect of learning across test trials, we only examined behaviors from the first test trials of sessions testing the knowing/acting mismatch (i.e., sessions 1 and 2) (Table B in S1 Appendix, Question 3) and the trials in which the orientation of the box initially changed in the session testing inhibition of a previously learned response (i.e., session 3) (Table B in S1 Appendix, Question 4). For these models, we included sex and age as predictors, and session (knowing/acting mismatch: 1 vs. 2; previously learned response: ‘orientation change number’, i.e., 1^st^ – 4^th^) as a covariate. We also included the behavioral measure (i.e., latency to enter or time spent inside the box) during the trial immediately before the given test trial as a covariate, to account for any baseline differences in behavior among individuals. For models testing the knowing/acting mismatch, this was the last familiarization trial of each session; for models testing inhibition of a previously learned response, this was the trial immediately before the box orientation changed. Models testing inhibition of a previously learned response were linear mixed effects models with elephant ID as a random effect. For models testing the knowing/acting mismatch, mixed effects models returned a ‘singular’ error due to negligible variation explained by the random effect, so we used linear models.

#### Change in performance across trials.

Lastly, we investigated whether performance changed over time (i.e., learning). For models investigating learning in the context of the knowing/acting mismatch (Table B in S1 Appendix, Question 5), we included all familiarization and test trials from sessions 1 and 2, excluding the first familiarization trial in session 1. We included trial number (1–4), trial type (familiarization vs. test), and the interaction between trial type and trial number as predictors, session (1 vs. 2) and box orientation (over vs. under) as covariates, and elephant ID as a random effect. For models investigating learning in the context of inhibiting a previously learned response (Table B in S1 Appendix, Question 6), we included all data from session 3. We numbered trials 1–4 within each box orientation (i.e., left, right, over, under) to determine whether performance improved when the elephant was given the same task in four consecutive trials. We also categorized box orientation as either horizonal (left or right) or vertical (over or under) to determine if there was a difference in behavior between box orientations with which elephants had never been familiarized using an opaque box (i.e., left and right), and those that they had (i.e., over and under). Models included box orientation (horizontal vs. vertical), trial number (1–4), and the interaction between box orientation and trial number as predictors, and elephant ID as a random effect. Lastly, for models investigating whether performance improved across trials in which elephants were presented with a randomized order of box orientations (i.e., session 4), we included all data from session 4 (Table B in S1 Appendix, Question 7). We numbered trials (randomized orientation) from 1–8 and included trial number as the predictor. The model with time spent inside the box as the dependent variable was a linear mixed effects model and included elephant ID as a random effect, but the model with latency to enter as the dependent variable returned a ‘singular’ error due to negligible variation explained by the random effect, so we used a linear model.

#### Inter-rater reliability.

We assessed inter-rater reliability between two coders for latency to enter, number of touches, and time spent inside the box using intraclass correlation coefficients (ICC), with the *irr* package [72].

## Results

### Prepotent responses

**Fig 2:**
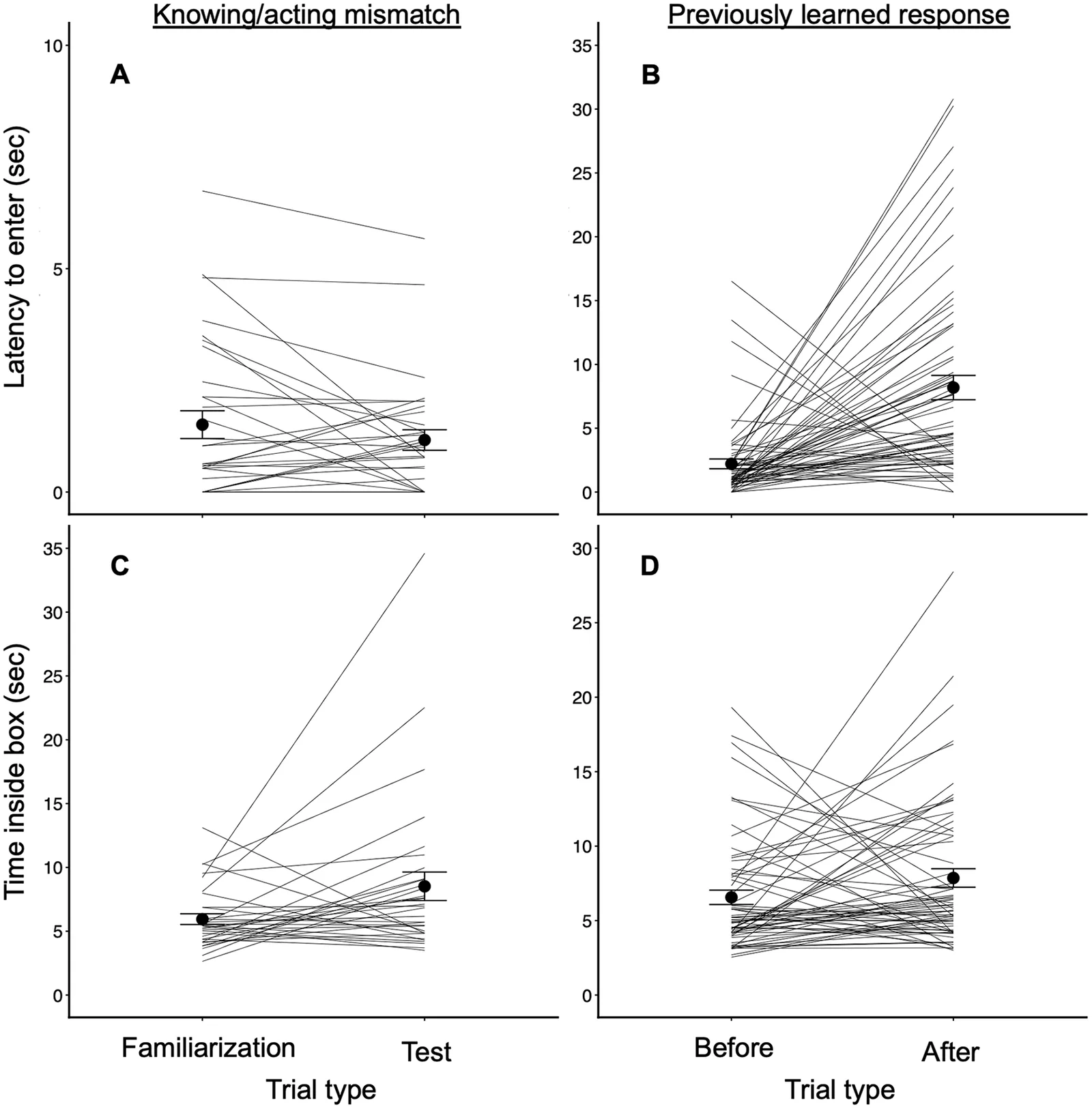
Prepotent Responses. **(A)** Latency to enter (sec) did not differ between the last familiarization and first test trial (*p* > 0.025) in sessions 1 and 2, but **(B)** was longer (*p* < 0.001) in trials in which the box orientation was changed for the first time (After) compared to the trial immediately preceding (Before) in session 3. **(C)** Time spent inside the box (sec) was greater (*p* = 0.013) in the first test trial compared to the last familiarization trial in sessions 1 and 2, but **(D)** did not differ before and after the box orientation changed in session 3 (*p* > 0.025). Points represent mean ± SE. Each line connects the two points between the behavior of a single elephant during either their **(A,C)** last familiarization trial and their first test trial during sessions 1 and 2 or **(B,D)** the trial in which the box orientation changed for the first time and the trial immediately previous in session 3. Therefore, each elephant is represented by two **(A,C)** or four lines **(B,D)**. Both dependent variables were log-transformed in the analysis to meet model assumptions, but raw data are presented here for clarity.

When investigating inhibitory control in relation to the knowing/acting mismatch (sessions 1 and 2), we found that elephants’ latency to enter did not differ between the last familiarization trial (mean ± SE = 1.51 ± 1.8 sec) and first test trial (1.16 ± 1.3 sec; *X*^*2*^ = 0.37, *p* = 0.54; Table B in S1 Appendix; Fig 2A); however, elephants spent more time with their trunk inside the box in the first test trial (8.52 ± 1.1 sec) compared to the last familiarization trial (5.94 ± 0.4 sec; *N* = 16 elephants, 64 trials; *X*^*2*^ = 5.89, *p* = 0.013; Fig 2C). When investigating inhibition of a previously learned behavior (session 3), we found that elephants had a longer latency to enter the box when the orientation first changed (8.18 ± 1.0 sec) compared to in the previous trial in which they had encountered the same box orientation for the fourth time (2.21 ± 0.4 sec; *N* = 16 elephants, 128 trials; *X*^*2*^ = 59.9, *p* < 0.001; Fig 2B); however, there was no difference in time spent inside the box (before orientation change: 6.56 ± 0.5 sec, after orientation change: 7.86 ± 0.6 sec; α = 0.025; *X*^*2*^ = 3.95, *p* = 0.047; Table B in S1 Appendix; Fig 2D). Latency to enter also increased in session 2 compared to session 1 (*X*^*2*^ = 10.9, *p* < 0.001), but there were no other relationships between behaviors and session (1 and 2) or orientation change number (all *p* > 0.025 [α]; Table B in S1 Appendix).

### Repeatability

In sessions 1 and 2, latency to enter (*R* = 0.257, SE = 0.097, CI (95%) = 0.068–0.440, *p* = 0.001) and the total time spent inside the box (*R* = 0.162, SE = 0.086, CI = 0.004–0.344, *p* = 0.005) were significantly repeatable. In session 3, latency to enter (*R* = 0.081, SE = 0.046, CI = 0–0.181, *p* = 0.002) and the total time spent inside the box (*R* = 0.233, SE = 0.079, CI = 0.082–0.382, *p* = 0.001) were significantly repeatable; however, note that the 95% confidence interval for the repeatability of latency to enter included zero. In session 4, time spent inside the box was significantly repeatable (*R* = 0.243, SE = 0.097, CI = 0.060–0.426, *p* = 0.001); however, latency to enter was not (*R* = 0, SE = 0.032, CI = 0–0.114, *p* = 1.00). We also note that all repeatability models had singular fits, suggesting low variance in behaviors among individuals.

### Age and sex differences

**Fig 3:**
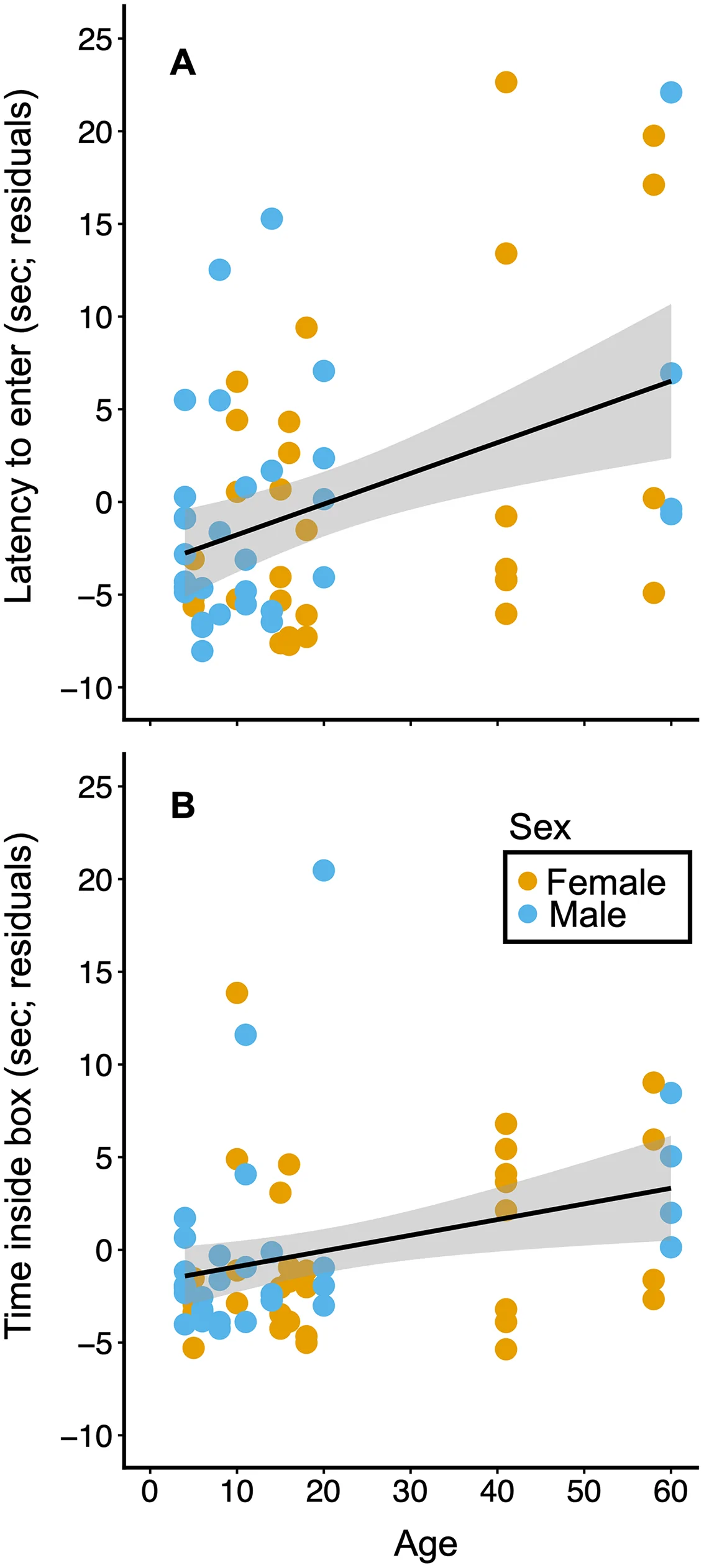
Age and Sex Differences. Age (years) was positively related to **(A)** the latency to enter (sec; *p* = 0.001) and **(B)** the time inside the box (sec; *p* = 0.003) during trials in which the box orientation changed for the first time in session 3. Data are represented as the residuals of the relationship between trials in which the box orientation was changed and the trial immediately previous, and therefore account for individual baseline differences in behavior. Each point represents a single trial; each elephant underwent four trials in session 3 in which the box orientation was changed. Thus, there are four points per elephant. The shaded areas represent the standard errors of the regression lines. Both dependent variables were log-transformed in analyses to meet model assumptions, but residuals of raw data are plotted here for clarity. Sex (orange = female, blue = male) was not related to either behavior (all *p* > 0.025).

When we investigated behaviors in the first test trials of sessions 1 and 2 (i.e., knowing/acting mismatch), we did not find any relationships between sex or age and latency to enter or time spent inside the box (*N* = 16 elephants, 32 trials; all *p* > 0.025; Table B in S1 Appendix; Fig A in S1 Appendix). However, when we investigated behaviors in the trials of session 3 in which the box orientation was changed for the first time (i.e., inhibition of a previously learned response), we found that age was positively related to both the latency to enter (*N* = 16 elephants, 64 trials; *X*^*2*^ = 10.39, *p* = 0.001; Fig 3A) and the time spent inside the box (*X*^*2*^ = 9.04, *p* = 0.003; Fig 3B), but there were no relationships with sex (all *p* > 0.025; Table B in S1 Appendix). In these models, we also included the behavior in the previous trial as a covariate. We found that, in session 3, the latency to enter the box in trials in which the box orientation changed was negatively related to the latency in the immediately preceding trials (*X*^*2*^ = 11.47, *p* < 0.001). However, plotting this relationship revealed that it was largely driven by four data points (out of 64 data points; Fig B in S1 Appendix). This covariate was not significantly related to behavior in any other model, and neither session number nor orientation change number were significantly related to any behavior in these models (all *p* > 0.025; Table B in S1 Appendix).

### Change in performance across trials

**Fig 4:**
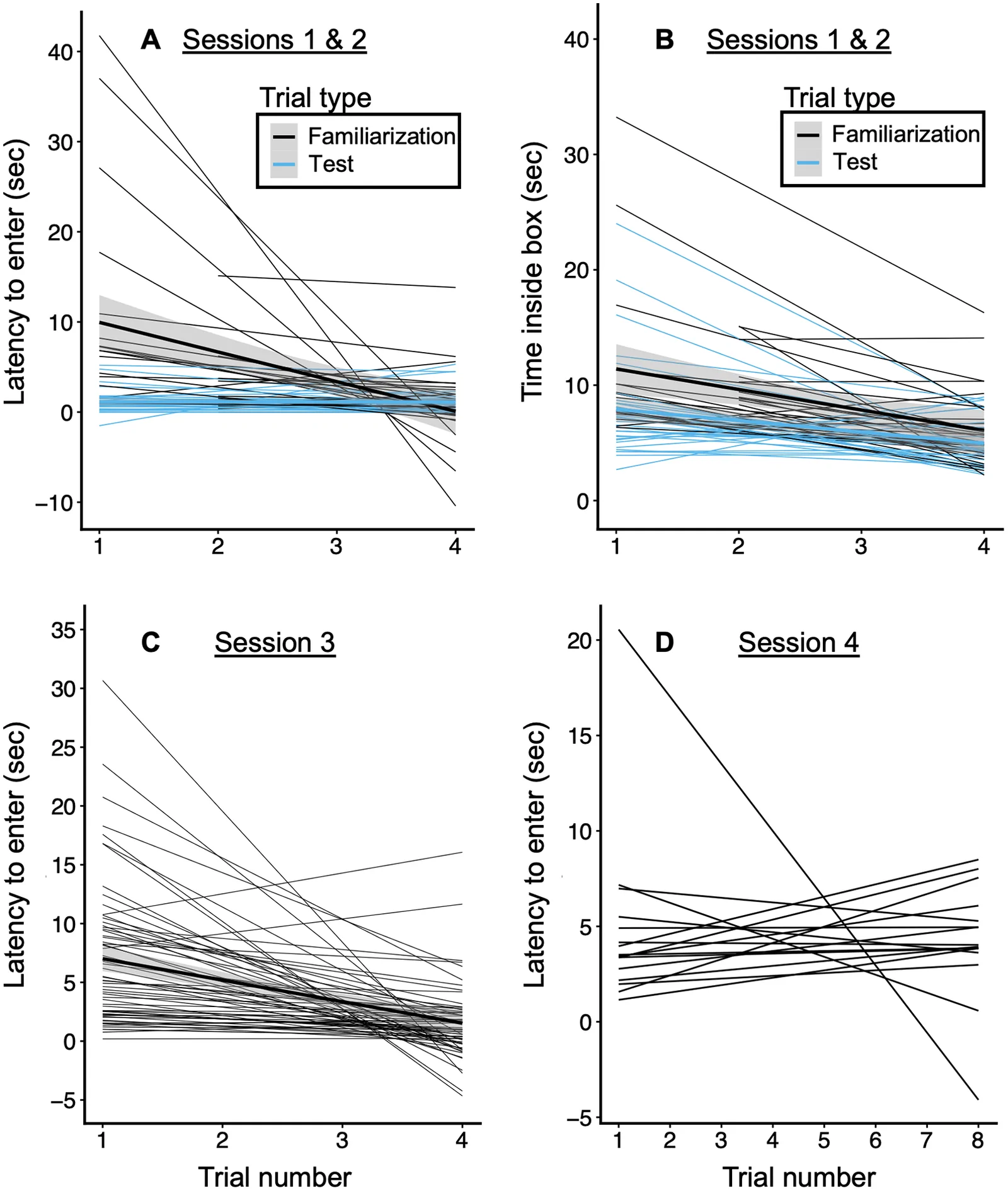
Change in Performance Across Trials. In sessions 1 and 2, **(A)** there was a significant interactive effect of trial number and trial type (black = familiarization; blue = test) on latency to enter (sec; *p* < 0.001) and **(B)** time spent inside the box (sec) decreased with trial number (*p* < 0.001) and was shorter in the test trials compared to familiarization trials (*p* < 0.001). In session 3, **(C)** latency to enter (sec) decreased across trials (within the same box orientation) (*p* < 0.001). In session 4, **(D)** there was no relationship between latency to enter (sec) and trial number (*p* > 0.025). In panels **A** and **B**, each thin line is the regression line for a single elephant for each trial type of sessions 1 and 2. Therefore, each elephant is represented by four lines. The first familiarization trial of session 1 was excluded. In panel **C**, each line is the regression line for a single elephant within one box orientation (either left, right, over, or under). Therefore, each elephant is represented by four regression lines. In panel **D**, each line is the regression line for a single elephant across the eight trials conducted. Therefore, each elephant is represented by one regression line. Thick lines are regression lines of all data; shaded areas represent the standard errors. All dependent variables were log-transformed in the analysis to meet model assumptions, but regression lines are based on raw data for clarity.

Overall, elephants performed well in the detour task and, across all trials (excluding the first familiarization trial), all individuals entered the box opening in less than one minute (latency to enter: max = 57.93 sec; mean ± SE = 3.68 ± 0.23 sec). In the context of the knowing/acting mismatch, we found evidence that elephants’ performance changed across trials. There was an interactive effect of trial number (1–4) and trial type on latency to enter (*N* = 16 elephants, 240 trials; *X*^*2*^ = 28.95; *p* < 0.001). Latency to enter decreased as trial number increased in familiarization trials, but did not change across test trials (Fig 4A). The time spent inside the box decreased with trial number (*X*^*2*^ = 33.87; *p* < 0.001; Fig 4B) and was shorter in test trials compared to the familiarization trials (*X*^*2*^ = 13.73; *p* < 0.001; Fig 4B), but there was no interaction (*X*^*2*^ = 3.49, *p* = 0.062; Table B in S1 Appendix). In addition, latency to enter was greater in session 2 compared to session 1 (*X*^*2*^ = 29.44; *p* < 0.001), but the time spent inside the box did not differ based on session (*X*^*2*^ = 1.74, *p* = 0.19; Table B in S1 Appendix). Both latency to enter and the time spent inside the box were greater when the box orientation was under compared to over (i.e., the elephant needed to reach under the box compared to over the top to enter it—all *X*^*2*^ ≥ 8.89; *p* ≤ 0.003; Table B in S1 Appendix).

When elephants were tested with the same box orientation for four consecutive trials (i.e., session 3), we found that, although latency to enter increased each time that the box orientation was changed (see *Prepotent responses*), it decreased across trials within the same orientation (*N* = 16 elephants, 256 trials; *X*^*2*^ = 29.16; *p* < 0.001; Fig 4C). Time spent inside the box did not differ across trials (*X*^*2*^ = 3.97, α = 0.025, *p* = 0.046; Table B in S1 Appendix; Fig C in S1 Appendix). Additionally, behaviors did not differ based on box orientation (all *p* > 0.025; Table B in S1 Appendix) and there were no interactive effects of box orientation and trial number on behaviors (all *p* > 0.025; Table B in S1 Appendix).

When elephants were tested with a random sequence of box orientations (i.e., session 4), we did not find evidence for a change in performance over time. Neither latency to enter nor time spent inside the box were related to trial number (*N* = 16 elephants, 128 trials; all *p* > 0.025; Table B in S1 Appendix; Fig 4D; Fig C in S1 Appendix).

### Investigating use of odor cues

No elephant reached directly for the open side of the box in the first trial of the third session (i.e., the session in which they encountered a completely novel box orientation), suggesting that they did not use odor cues to directly find the opening before first interacting with the box.

### Inter-rater reliability

Co-author G.S. and the independent coder had excellent reliability [73] for the latency to enter (ICC(A,1) = 0.979, 95% CI = 0.966–0.986, F(159,48.5) = 107, *p* < 0.001), number of touches (ICC(A,1) = 0.958, 95% CI = 0.943–0.969, F(159,140) = 48.3, *p* < 0.001), and time spent inside the box (ICC(A,1) = 0.914, 95% CI = 0.870–0.941, F(159,63.3) = 24.5, *p* < 0.001).

## Discussion

Here, we investigated inhibitory control in Asian elephants using a classic detour paradigm [10]. To date, only one study has investigated the inhibitory control of Asian elephants, and found that they performed poorly on the A-not-B task [6]. Overall, we found that elephants performed well in the detour task and that, in general, individual behaviors had low but significant repeatability. Once elephants learned that the box around which they needed to detour contained food (i.e., the first familiarization trial), all individuals entered the opening of the box in under one minute, regardless of box orientation or opacity. When faced with a transparent surface after being trained to detour around an opaque barrier (i.e., the ‘knowing/acting mismatch’), elephants did not show any difference in latency to enter the opening, and continued to quickly detour around the transparent barrier. This may provide evidence that elephants exhibit very strong inhibitory control; however, it may also indicate that the transparent surface did not elicit a strong prepotent response. In contrast, we did find that changing the orientation of the box elicited a prepotent response. However, elephants were subsequently able to inhibit the response to reach toward a previously rewarded side of the box and improve detour performance across trials—providing evidence for strong inhibitory control. We also found that task performance varied across individuals, and that some of this variation was explained by age. Overall, the evidence for strong inhibitory control in Asian elephants that we found in this study agrees with the general positive relationship between brain size and inhibitory control ability that is found across other taxa [6].

### Overcoming the knowing/acting mismatch

If elephants succumb to the impulse to reach straight for a reward [i.e., “knowing/acting mismatch,” 1,10], we would expect that, after being trained to detour around a solid opaque box, their detouring would become slower when faced with a transparent barrier with front-facing holes. However, in sessions 1 and 2, elephants’ latency to enter the box did not differ between the final encounter with the opaque box and the first encounter with the transparent box. Elephants then continued to detour quickly and consistently across the subsequent test trials (Fig 4A), despite the cues from the transparent box. It is possible that this indicates strong inhibitory control to inhibit the impulse to reach straight forward toward the front-facing visual and olfactory cues (i.e., ‘acting’), in favor of continuing an effective behavior (i.e., ‘knowing’). Indeed, our results are similar to other detour-reaching studies which have found that great apes [6] and corvids [7] performed flawlessly (i.e., never touched the barrier) or near-flawlessly in detouring around a transparent surface after being trained with a similar opaque barrier. Recent studies suggest that elephants have similar cognitive abilities to great apes and corvids [reviewed in 39,74]; thus, this study may represent another example of convergent cognitive evolution among these species.

However, because we did not find a ‘characteristic error pattern’ [23] after introducing the transparent barrier, we cannot disregard the simpler explanation that an impulse was never evoked. Although Asian elephants do respond to visual cues [e.g., 75–77] and thus should be able to perceive the differences between the opaque and transparent boxes, they often prioritize other senses—such as olfaction, audition, and touch—over vision [39]. Because of this, we also attempted to divert olfactory cues by putting holes in the front face of the box. Although we did not expect that the olfactory cues coming from these holes would be stronger than those coming from the open side, we expected that the olfactory cues coming from two directions (i.e., from the opening and from the front face) would slow elephants’ detour response. However, these cues may not have been strong enough to elicit a prepotent response. More importantly, elephants appeared not to rely on olfactory cues to find the opening. Indeed, in the first trial of session 3, in which elephants were presented with a novel box orientation for the first time, no elephant directly entered the opening—suggesting that they did not use olfactory cues to locate the opening before first touching the box. Taken together, we think that it is more likely that elephants’ strong performance in sessions 1 and 2 was driven by their use of a habitual motor response, rather than the inhibition of an impulse evoked by visual or olfactory cues.

Interestingly, in sessions 1 and 2, elephants spent more time with their trunks inside the box when they were first exposed to the transparent box compared to the previous familiarization trial. This difference in behavior suggests that, even if a prepotent response was not evoked, elephants still appear to have been able to perceive the difference between the opaque and transparent boxes. Although this increase in time spent inside the transparent box is not related to inhibitory control—because elephants had already detoured around the barrier—this behavior could indicate an increase in exploratory behavior when interacting with the transparent box. In other species, exposure to novel stimuli leads to increased exploration and curiosity [e.g., 78,79], so elephants may have been spending time exploring the novel stimulus (i.e., a transparent surface) or situation (e.g., seeing their trunk behind a transparent surface). Indeed, time spent inside the box subsequently decreased across test trials (Fig 4B), suggesting that, as may be expected, exploratory behavior decreased as elephants became accustomed to the new surface. Unlike sessions 1 and 2, time spent inside the box did not change across trials in either session 3 or 4, likely because elephants already had experience with the transparent surface. Although we cannot discount the possibility that the change in behavior we observed in sessions 1 and 2 may have been due to differences between the opaque and transparent boxes other than transparency, we think it is unlikely. Specifically, the box exteriors differed in texture (i.e., the exterior of the opaque box was spray-painted), but box interiors were identical. However, if exterior differences did affect elephant behavior, we think it would be more likely to cause a change in behavior in the latency to enter—where elephants needed to interact with the box exterior to find the opening—but it did not. In addition, although we initially considered time spent inside the box as a measure of motor control, it is unlikely that motor control differences explain the observed behavior, as surface transparency is unlikely to affect an elephant’s motor control.

### Inhibition of a previously learned response

To test elephants’ capacity to inhibit a previously learned response, elephants first experienced the detour box with its opening in a certain orientation, and then we changed the orientation to measure how quickly elephants could learn to enter their trunk into a novel open side [13,14]. We found that, each time elephants were presented with a novel box orientation in session 3, their latency to enter the box—our main measure of inhibitory control—increased (Fig 2B). Similarly, latency to enter increased in session 2—when elephants first experienced a novel box orientation (i.e., either under or over)—compared to session 1. This ‘characteristic error pattern’ [23] of a slower latency to enter suggests that switching box orientations evoked a prepotent response, making it a valid measure of inhibitory control. Then, when elephants were presented with the same box orientation across multiple trials (i.e., in sessions 1, 2, and 3), latency to enter decreased across trials (Figs 4A and 4C). This demonstrates that elephants quickly learned to inhibit a previously rewarded, but no longer effective, motor response and flexibly changed their behavior to detour around the most recent box orientation—suggesting strong inhibitory control.

Elephants appeared to improve their performance in this detour task by quickly searching using short-range cues and performing based on a habitual motor response, instead of using long-range visual or olfactory cues. At first, we thought that elephants may use olfactory cues coming from the open face of the box to directly target the opening before interacting with the box. However, this is unlikely because, in session 3 when the opening was in a novel position (i.e., left or right) for the first time, no elephant ever went directly to the opening. Furthermore, in trials in which the box orientation was different from that in the previous trial (i.e., the first familiarization trial of session 2, the first time each orientation was changed in session 3, and every trial of session 4), elephants directly entered the opening (i.e., latency to enter = 0 sec) in only 8 out of 208 trials. This suggests that elephants likely did not use cues to find the opening before first touching the box, but rather began searching after they had touched it. It is possible that this search behavior is the result of the elephants’ propensity to use tactile cues exclusively in this context, or their use of tactile information prior to seeking olfactory cues; in other words, once the elephants make contact with the box or get close to it, they may then initiate their olfactory search behavior, leading to exploration for the box opening. In contrast, in trials in which the box orientation was the same as in the previous trial (including familiarization and test trials), elephants went directly to the opening significantly more frequently (70 out of 416 trials; two-sample test of proportions: *X*^*2*^ = 20.2, *p* < 0.001), suggesting that they had learned a habitual response. However, when elephants could not rely on a habitual response, they were still able to access the opening by quickly searching using tactile and/or short-range olfactory cues. Indeed, when the sequence of box orientations was random (i.e., session 4), most elephants appeared to have reached ‘peak’ performance in inhibiting the previous response and quickly searching for the new opening, with a quick latency to enter (mean ± SE = 4.52 ± 0.40 sec) that did not significantly vary across trials (Fig 4D).

### Age and sex differences

Age was related to both latency to enter and time spent inside the box in trials in which elephants needed to inhibit a previously learned response (i.e., session 3; Fig 3). It is unlikely that these relationships with age are due to weaker motor control in older individuals because the models investigating age differences also included the behavior in the previous trial (i.e., before the box orientation was changed) as a covariate, which should account for baseline differences in trunk speed between individuals. This suggests that older elephants exhibited different behaviors in response to the change in box orientation, even after considering that older elephants may move their trunks more slowly compared to younger elephants, in general. First, we found that age was positively related to the latency to enter the box in trials in which the box orientation was initially changed (i.e., 1^st^, 5^th^, 9^th^, and 13^th^ trials of session 3). This suggests that it may have been more difficult for older individuals, compared to younger individuals, to inhibit a previously learned response. This is consistent with studies in other species that have found that inhibitory control declines in old age [e.g., humans, [80,81], marmosets, [50], dogs, [21], but see [58], which found that older mosquitofish had greater inhibitory control compared to younger individuals]. Although it is hypothesized that elephants may not succumb to a cognitive decline [reviewed in 47], our results suggest that inhibitory control may be at least one aspect of cognition that decreases with age in elephants. However, our study had a relatively small sample size that was weighted towards younger elephants. A larger distribution of ages may allow for the investigation of more nuanced patterns of relationships between age and cognition [49,50].

Time spent inside the box also increased with age when elephants needed to inhibit a previously learned behavior (Fig 3B). This may indicate that older elephants were more exploratory than younger elephants when faced with something new—in this case, the changes in box orientation. Indeed, Kendal et al. [82] found that older callitrichid monkeys were more exploratory when presented with multiple novel extractive foraging tasks compared to younger individuals. However, studies in other species have found the opposite—that juveniles are more attracted to novelty than adults [83–85]. In addition, it has been hypothesized that a decrease in exploratory behavior with age may be adaptive across species [86]. Results from one study that investigated whether semi-captive Asian elephants would walk over a novel surface when instructed by a mahout are consistent with these latter studies—older elephants tended to be less likely to cross the novel surface compared to younger elephants [87]. However, in wild Asian elephants, age does not appear to be related to exploratory diversity in an innovative problem-solving task [32] or to exploration of novel objects [88]. Our study is the first, to our knowledge, to find a positive relationship between a measure of exploration and age in elephants; however, further work with larger samples and greater variation in life histories is needed to better understand elephant exploratory behavior.

We did not find any differences between sexes in any behavioral measure. The lack of sex differences in inhibitory control is consistent with some studies [e.g., robins, [19], dogs, [22], mouse lemurs, 89]; however, more studies have found that females have greater inhibitory control compared to males [e.g., humans, [56,57], baboons, [60], rhesus macaques, [55], dogs, [59], mosquitofish, 58]. We originally predicted that inhibitory control would differ between sexes because female elephants live in fission-fusion societies—which are associated with high inhibitory control [5]—whereas males live solitarily or in small bachelor groups [43,46]. However, because males stay with the herd until sexual maturity [~10–15 years, 43], we may predict that a sex difference in inhibitory control would only be apparent in mature elephants. Our study included only four male elephants who were ≥10 years old. Therefore, we may have found sex differences if we had tested more older adult males. It would also be interesting to investigate whether there is a relationship between inhibitory control and sex in the wild, where social and environmental pressures on the expression of cognition may be stronger than in captivity.

## Conclusions

To our knowledge, this study provides the first evidence for inhibitory control in Asian elephants, and improves our understanding of the cognition of this unique species. Recent studies have provided insight into the complex cognitive abilities of Asian elephants [31–35,61,75,76,90], and strong inhibitory control may be a cognitive mechanism that underlies or relates to some of these abilities, such as problem-solving [e.g., 36,38]. Strong inhibitory control may also be necessary for elephants’ complex sociality, which includes fission-fusion dynamics [5] and cooperation [2,37]. In addition, understanding inhibitory control may aid in the conservation of this endangered species [91]. One of the biggest threats to Asian elephants is human-elephant conflict (HEC), which arises when elephants enter into crop fields—often by navigating around human-made barriers (e.g., electric fences)—to eat crops [92]. Understanding how elephants interact with and learn to detour around different types of barriers in controlled inhibitory control experiments may help us understand how they avoid or circumvent human-installed obstacles in the wild. This may, in turn, inform the development of more effective HEC mitigation strategies that are tailored to how elephants navigate the world [93].

Here, we tested elephants using a classic detour paradigm, with the goal of producing data that can be interpreted alongside the numerous studies that have used this paradigm with other species [10], to further our knowledge of how inhibitory control may have evolved across taxa. However, this is only the beginning of understanding elephant inhibitory control. Future studies should focus on using methods that continue to consider elephants’ tactile, olfactory, and/or auditory senses [39]. One aspect that we could not completely account for in this study was the potential relationships between personality traits and inhibitory control. Further work is needed to explicitly quantify whether aspects of personality—such as boldness, proactivity, or exploration [16–18]—may underlie individual differences in inhibitory control. Finally, in this study, we found that age was negatively related to the ability to inhibit a previously learned response; however, more work is needed to understand what other demographic or social factors may drive individual variation in inhibitory control.

## Supporting information

S1 Appendix All Supporting Tables and Figures. Includes two supporting tables and three supporting figures.
(DOCX)

S1 Video Familiarization Trial. An elephant (ThongChai) interacting with the familiarization (opaque) box. The box is in the “under” orientation and the reward is half of an apple. Video was taken on a Sony FDR-AX100 camcorder.
(MP4)

S2 Video Testing the Knowing/Acting Mismatch. An elephant (ThongChai) interacting with the test (transparent) box. The box is in the “under” orientation and the reward is half of an apple. Note that this is the same elephant as in Video 1, and this test trial occurred immediately after familiarization trials with the “under” orientation. Video was taken on a Sony FDR-AX100 camcorder.
(MP4)

S3 Video Testing Inhibition of a Previously Learned Behavior. An elephant (Malini) interacting with the test box positioned in the “left” orientation. Note that this is her first interaction with the box in this orientation. This trial immediately follows four trials of the box in the “right” orientation. The reward is half of an apple. Video was taken on a Sony FDR-AX100 camcorder.
(MP4)
