# Supplementary material for: Asian elephants (Elephas maximus) demonstrate inhibitory control in a detour task
Source: PLoS One. 2026 Sep 9;21(9):e0356549. doi: 10.1371/journal.pone.0356549 (PMC13557342; doi:10.1371/journal.pone.0356549)
Supplement: Supplementary file 1 [file pone.0356549.s001.docx]

**Supporting information**

**S1 Appendix**

Asian elephants (*Elephas maximus*) demonstrate inhibitory control in a detour task

Sydney F. Hope^1*^, Sarah L. Jacobson^1,2,3^, Sangpa Dittakul^4^, Gillian Schmitt^1^, Mananya Pla-ard^4^, Marnoch Yindee^5^, Taweepoke Angkawanish^6^, Joshua M. Plotnik^1,2*^

^1^ Department of Psychology, Hunter College, City University of New York, New York, New York, United States of America

^2^ Psychology Ph.D. Program, CUNY Graduate Center, City University of New York, New York, New York, United States of America

^3^ Department of Psychology, Saint Xavier University, Chicago, Illinois, United States of America

^4^ Golden Triangle Asian Elephant Foundation, Chiang Saen, Chiang Rai, Thailand

^5^ Akkhraratchakumari Veterinary College, Walailak University, Nakhon Si Thammarat, Thailand

^6^ National Elephant Institute, Forest Industry Organization, Lampang, Thailand

*** Corresponding authors

Email: sydney.hope@gmail.com (SFH), ORCID 0000-0002-3711-8593;

Email: joshua.plotnik@gmail.com (JMP), ORCID 0000-0002-7597-8818

**Table A.** Names, sexes, and ages of elephants who participated in the current study.

| **Name** | **Sex** | **Age (years)** |
| --- | --- | --- |
| Baiboon | F | 5 |
| Somsri | F | 10 |
| Pachee | F | 15 |
| Nammei | F | 16 |
| Malini | F | 18 |
| Khod | F | 41 |
| Phumpuong | F | 41 |
| Wandee | F | 58 |
| Butoo | M | 4 |
| Khomsan | M | 4 |
| Sanlan | M | 6 |
| Boon | M | 8 |
| TongKham | M | 11 |
| ThongChai | M | 14 |
| Sapriwan | M | 20 |
| Boonsaen | M | 60* |

*Boonsaen’s exact age was unknown, but his mahout confirmed he was at least 60 years old; we used 60 as his age for modelling purposes.

**Table B. All Model Results.** Models are organized by question and follow the order used in the *Results* section of the manuscript. For each model, we specify the type of model used, any necessary transformations that were used to meet model assumptions, the data included, the fixed and random effects, and the sample size. We present model estimates (B) and standard error (SE) from the *summary* function in R. Test statistics and *p*-values are also reported using the *summary* function when possible; when *p*-values were not included in the output of *summary* (i.e., linear mixed effects models), we used the *Anova* function of the *car* package, with type III sums of squares. In each case, we report the test statistic (either *t* or *X^2^*) that is provided by R for each type of model. We used a Bonferroni correction because all models were conducted twice (i.e., once for latency to enter and once for time spent inside box). Therefore, we set significance at α = (0.05/2) = 0.025. Variables in brackets indicate the reference level for categorical variables. The R script used to carry out all analyses can be found on Figshare (https://doi.org/10.6084/m9.figshare.31855732).

| **PREPOTENT RESPONSES** | | | | | | | | |
| --- | --- | --- | --- | --- | --- | --- | --- | --- |
| ***Question 1:*** *Does behavior differ when elephants were presented with the transparent box compared to the opaque box (i.e., do elephants succumb to the knowing/acting mismatch)?*  *Data included:* the last familiarization trial and first test trial of sessions 1 and 2 for each elephant  *N =* 16 elephants; 64 trials | | | | | | | | |
|  | **Dependent variable** | | | | | | | |
|  | **Latency to enter** | | | | **Time spent inside box** | | | |
|  | linear mixed effects model  log-transformation | | | | linear mixed effects model  log-transformation | | | |
| **Fixed effects** | B | SE | *X^2^* | *p* | B | SE | *X^2^* | *p* |
| Trial type (familiarization vs. test) [test] | -0.079 | 0.13 | 0.37 | 0.54 | 0.27 | 0.11 | 6.22 | **0.013** |
| Session (1 vs. 2) | 0.43 | 0.13 | 10.9 | **<0.001** | -0.20 | 0.11 | 3.61 | 0.057 |
| **Random effects** |  |  | Var | SD |  |  | Var | SD |
| Elephant ID |  |  | 0.028 | 0.17 |  |  | 0.020 | 0.14 |
| Residual |  |  | 0.27 | 0.52 |  |  | 0.18 | 0.43 |
| ***Question 2:*** *Does behavior differ when presented with a new box orientation (i.e., can elephants inhibit a previously learned behavior)?*  *Data included:* trials from session 3 in which the box orientation changed for the first time (i.e., trials 1, 5, 9, and 13), and their immediately preceding trials; the immediately preceding trial to the first trial of session 3 is the last trial of session 2  *N =* 16 elephants; 128 trials | | | | | | | | |
|  | **Latency to enter** | | | | **Time spent inside box** | | | |
|  | linear mixed effects model  log-transformation | | | | linear mixed effects model  log-transformation | | | |
| **Fixed effects** | B | SE | *X^2^* | *p* | B | SE | *X^2^* | *p* |
| Trial type (before orientation change vs. after) [before] | -0.98 | 0.13 | 59.9 | **<0.001** | -0.16 | 0.082 | 3.95 | 0.047 |
| Orientation change number (1-4; i.e., number of times that orientation was changed) | 0.053 | 0.057 | 0.89 | 0.35 | -0.049 | 0.036 | 1.82 | 0.18 |
| **Random effects** |  |  | Var | SD |  |  | Var | SD |
| Elephant ID |  |  | 0.032 | 0.18 |  |  | 0.050 | 0.22 |
| Residual |  |  | 0.51 | 0.72 |  |  | 0.21 | 0.46 |
| **AGE AND SEX DIFFERENCES** | | | | | | | | |
| ***Question 3:*** *Does behavior in reaction to the transparent surface (i.e., knowing/acting mismatch) differ between sexes or across ages?*  *Data included:* the first test trial of sessions 1 and 2; the last familiarization trial of each session is used as a covariate to account for individual differences in behavior  *N =* 16 elephants; 32 trials | | | | | | | | |
|  | **Latency to enter** | | | | **Time spent inside box** | | | |
|  | linear model^a^  log-transformation | | | | linear model^a^  log-transformation | | | |
| **Fixed effects** | B | SE | *t* | *p* | B | SE | *t* | *p* |
| Sex [male] | 0.018 | 0.16 | 0.11 | 0.91 | 0.12 | 0.19 | 0.62 | 0.54 |
| Age | 0.0064 | 0.0048 | 1.32 | 0.20 | 0.01 | 0.01 | 2.2 | 0.036 |
| Session (1 vs. 2) | 0.28 | 0.17 | 1.70 | 0.10 | -0.33 | 0.18 | -1.9 | 0.068 |
| Behavior in last familiarization trial | 0.11 | 0.053 | 2.01 | 0.054 | -0.01 | 0.04 | -0.3 | 0.75 |
| ***Question 4:*** *Does behavior in reaction to changing box orientations (i.e., overcoming a previously learned behavior) differ between sexes or across ages?*  *Data included:* trials from session 3 in which the box orientation changed for the first time; immediately preceding trials to these trials are used as covariates to account for individual differences in behavior  *N =* 16 elephants; 64 trials | | | | | | | | |
|  | **Latency to enter** | | | | **Time spent inside box** | | | |
|  | linear mixed effects model  log-transformation | | | | linear mixed effects model  log-transformation | | | |
| **Fixed effects** | B | SE | *X^2^* | *p* | B | SE | *X^2^* | *p* |
| Sex [male] | 0.27 | 0.22 | 1.53 | 0.22 | 0.11 | 0.12 | 0.87 | 0.35 |
| Age | 0.019 | 0.0060 | 10.39 | **0.0013** | 0.010 | 0.0034 | 9.04 | **0.0026** |
| Orientation change number | 0.061 | 0.082 | 0.55 | 0.46 | -0.098 | 0.047 | 4.41 | 0.036 |
| Behavior in previous trial | -0.11 | 0.032 | 11.47 | **<0.001** | 0.0041 | 0.015 | 0.078 | 0.78 |
| **Random effects** |  |  | Var | SD |  |  | Var | SD |
| Elephant ID |  |  | 0.040 | 0.20 |  |  | 0.0096 | 0.098 |
| Residual |  |  | 0.52 | 0.72 |  |  | 0.17 | 0.42 |
| **CHANGE ACROSS TRIALS (LEARNING)** | | | | | | | | |
| ***Question 5:*** *Does performance change across trials that test the knowing/acting mismatch?*  *Data included:* all trials in sessions 1 and 2, except the first familiarization trial of session 1  *N =* 16 elephants; 240 trials | | | | | | | | |
|  | **Latency to enter** | | | | **Time spent inside box** | | | |
|  | linear mixed effects model  log-transformation | | | | linear mixed effects model  log-transformation | | | |
| **Fixed effects** | B | SE | *X^2^* | *p* | B | SE | *X^2^* | *p* |
| Trial number | -0.40 | 0.054 | 56.51 | **<0.001** | -0.21 | 0.036 | 33.87 | **<0.001** |
| Trial type (familiarization vs. test) [test] | -1.53 | 0.20 | 57.96 | **<0.001** | -0.50 | 0.13 | 13.73 | **<0.001** |
| Session (1 vs. 2) | 0.42 | 0.077 | 29.44 | **<0.001** | -0.067 | 0.051 | 1.74 | 0.19 |
| Box orientation (over vs. under) [under] | 0.22 | 0.075 | 8.89 | **0.003** | 0.17 | 0.050 | 11.67 | **<0.001** |
| Trial number X Trial type [test] | 0.38 | 0.071 | 28.95 | **<0.001** | 0.088 | 0.047 | 3.49 | 0.062 |
| **Random effects** |  |  | Var | SD |  |  | Var | SD |
| Elephant ID |  |  | 0.056 | 0.24 |  |  | 0.030 | 0.17 |
| Residual |  |  | 0.33 | 0.58 |  |  | 0.15 | 0.39 |
| ***Question 6:*** *Does performance change across trials that test inhibition of a previously learned response?*  *Data included:* all trials in session 3  *N =* 16 elephants; 256 trials | | | | | | | | |
|  | **Latency to enter** | | | | **Time spent inside box** | | | |
|  | linear mixed effects model  log-transformation | | | | linear mixed effects model  log-transformation | | | |
| **Fixed effects** | B | SE | *X^2^* | *p* | B | SE | *X^2^* | *p* |
| Box orientation (horizontal vs. vertical) [vertical] | -0.07 | 0.22 | 0.11 | 0.74 | -0.25 | 0.13 | 3.61 | 0.057 |
| Trial number^b^ | -0.30 | 0.056 | 29.16 | **<0.001** | -0.067 | 0.034 | 3.97 | 0.046 |
| Box orientation [vertical] X trial number | 0.030 | 0.079 | 0.14 | 0.70 | -0.022 | 0.048 | 0.21 | 0.65 |
| **Random effects** |  |  | Var | SD |  |  | Var | SD |
| Elephant ID |  |  | 0.044 | 0.21 |  |  | 0.064 | 0.25 |
| Residual |  |  | 0.51 | 0.71 |  |  | 0.18 | 0.43 |
| ***Question 7:*** *Does performance change across trials in which box orientation is constantly changing?*  *Data included:* all trials in session 4  *N =* 16 elephants; 128 trials | | | | | | | | |
|  | **Latency to enter** | | | | **Time spent inside box** | | | |
|  | linear model^a^  log-transformation | | | | linear mixed effects model  log-transformation | | | |
| **Fixed effects** | B | SE | *t* | *p* | B | SE | *X^2^* | *p* |
| Trial number^c^ | <0.001 | 0.028 | 0.022 | 0.98 | -0.0026 | 0.016 | 0.026 | 0.87 |
| **Random effects** |  |  |  |  |  |  | Var | SD |
| Elephant ID |  |  |  |  |  |  | 0.056 | 0.24 |
| Residual |  |  |  |  |  |  | 0.18 | 0.42 |

^a^Linear mixed effects models were singular (R warning ‘isSingular’), which means that little to no variance was explained by random effects; in these cases, we used a linear model instead. In all cases, qualitative results did not differ between model types.

^b^In session 3, trials are numbered 1-4, separately for each orientation

^c^In session 4, trials are numbered 1-8

**Figure A.** Age and sex (orange = female; blue = male) were not related to **(1)** the latency to enter the transparent box or **(2)** time spent inside the box (*p* > 0.025) in sessions 1 and 2. Data are represented as the residuals between the last familiarization and first test trial of sessions 1 and 2, and therefore account for individual baseline differences in behavior. Each point represents one elephant in either session 1 or 2; thus, there are two points per individual. Both dependent variables were log-transformed in the analysis to meet model assumptions, but residuals of raw data are plotted here for clarity.

**Figure B.** Latency to enter (sec) in the trials in session 3 in which the box orientation changed (i.e., ‘after orientation change’) was negatively related to the latency to enter in the trial immediately previous (i.e., ‘before orientation change’; *p* < 0.001). Each point represents one trial in session 3 in which the box orientation changed; therefore, each elephant is represented by four points. The shaded areas around the regression line represent the standard error. The dependent variable was log-transformed in the analysis to meet model assumptions, but raw data are plotted here for clarity. Note that the negative relationship is largely driven by four points (out of 64). These four data points were from Boonsaen (when the box was switched from ‘under’ to ‘over’), Butoo (when the box was switched from ‘right’ to ‘under’), Khomsan (when the box was switched from ‘left’ to ‘over’), and Phumpuong (when the box was switched from ‘right’ to ‘under’).

**Figure C.** Time spent inside the box (sec) did not change across trials in **(1)** session 3 or **(2)** session 4 (all *p* > 0.025). For session 3, each line is the regression line for a single elephant within one box orientation (either left, right, over, or under). Therefore, each elephant is represented by four regression lines. For session 4, each line is the regression line for a single elephant across the eight trials conducted. Therefore, each elephant is represented by one regression line. All dependent variables were log-transformed in the analyses to meet model assumptions, but regression lines based on raw data are presented here for clarity.
